# A Rare Case of Aphasia Caused by Delayed Epidural Abscess 6 Years after Cranioplasty

**DOI:** 10.3390/diagnostics12092040

**Published:** 2022-08-24

**Authors:** Jae Ho Kim, Dae Kyun Kim, Seok Won Kim

**Affiliations:** Department of Neurosurgery, College of Medicine, Chosun University, 365 Pilmun-daero, Dong-gu, Gwangju 61453, Korea

**Keywords:** infection, aphasia, cranioplasty

## Abstract

Cranioplasty following decompressive craniectomy for severe head trauma or stroke encompasses various cranial reconstruction techniques that use autograft or allograft materials. It not only provides protection and cosmetic benefits to the brain but also facilitates neurological function. One of the most important and undesirable complications of cranioplasty is graft infection, which usually develops within several days to months. Here, we report the case of a 46-year-old man who was admitted with aphasia caused by a delayed epidural abscess 6 years after cranioplasty. The possible pathophysiological mechanisms of this rare entity are discussed along with a review of the relevant literature.

##  

**Figure 1:**
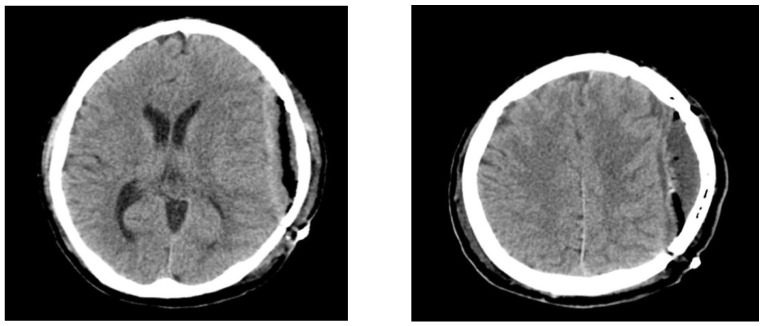
Brain computed tomography scans at 3 months after cranioplasty demonstrate air bubbles and epidural fluid collection. Although cranioplasty is a relatively simple procedure, it carries a substantial risk of postoperative infection or foreign body reaction mimicking infection [1,2] Postoperative infections are usually observed several days to months after cranioplasty operations, and delayed infections presenting 6 years after cranioplasty, as seen in our case, are rare. To the best of our knowledge, no studies have reported aphasia caused by delayed epidural abscess after cranioplasty. A 46-year-old man was admitted to our emergency room (ER) with a complaint of difficulty speaking that started 3 days prior. Left frontotemporoparietal (FTP) craniectomy and hematoma removal were performed for acute subdural hematoma 6 years prior. At 3 months after the decompressive craniectomy, cranioplasty was performed to repair the skull defect using cryopreserved autologous bone. Computed tomography (CT) at 3 months post-cranioplasty revealed postoperative air bubbles and epidural fluid collection (Figure 1).

**Figure 2:**
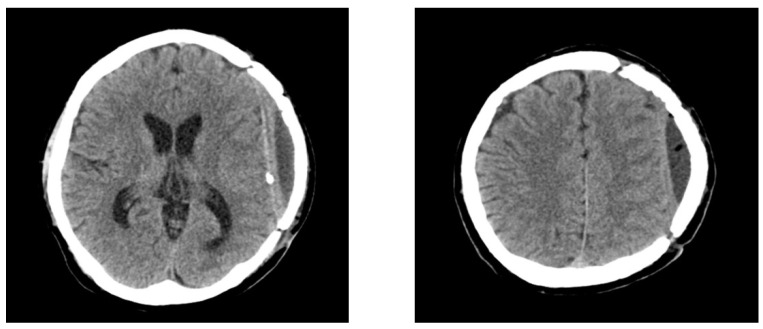
Brain computed tomography scans at 8 months after cranioplasty reveal improvement in the air bubbles and epidural fluid collection. However, the patient did not require any additional procedure due to the absence of neurological abnormalities, and a follow-up CT scan performed 8 months after the cranioplasty demonstrated decreased epidural fluid collection without midline shift (Figure 2).

**Figure 3:**
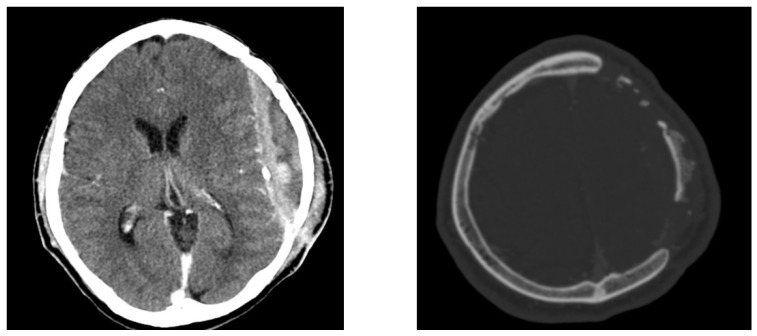
Brain contrast-enhanced computed tomography images show erosion of the bone flap and rim enhancement of the fluid collection. Physical examination did not reveal any tenderness, swelling, or wound dehiscence at the time of admission to the ER. However, squeezing of the left temporal incision site of the previous cranioplasty surgery caused the release of a small amount of purulent discharge. On neurological examination, the patient had a clear mental status and no neurological deficits; however, he was aphasic and murmured only simple words. On admission to the ER, his body temperature was normal (36.6 °C). Laboratory examination of infection markers revealed normal erythrocyte sedimentation rate (ESR), C-reactive protein (CRP) level, and white blood cell (WBC) count. The purulent discharge culture was negative for bacteria. Brain CT demonstrated an organized epidural abscess and resorption of the autologous implanted bone (Figure 3).

**Figure 4:**
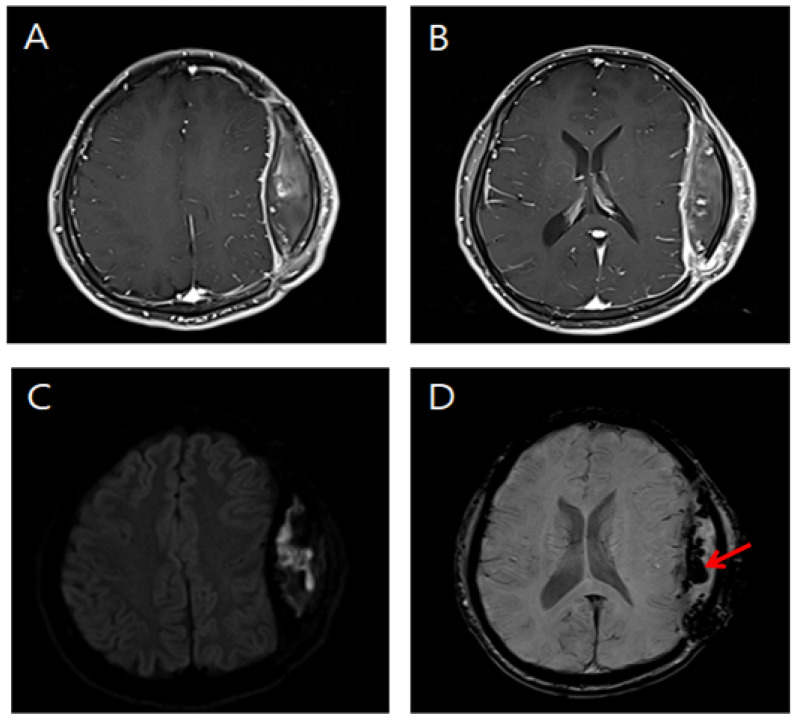
Brain magnetic resonance images of the patient. (**A**,**B**) Post-contrast T1-weighted images show rim enhancement of the fluid collection. (**C**) Diffusion-weighted image of the epidural fluid collection demonstrates restricted diffusion. (**D**) Susceptibility-weighted image of the epidural fluid collection reveals multiple punctate hypointensities secondary to hemorrhage (arrow). Brain magnetic resonance imaging showed a mass effect caused by a hemorrhagic component and epidural abscess (Figure 4).

**Figure 5:**
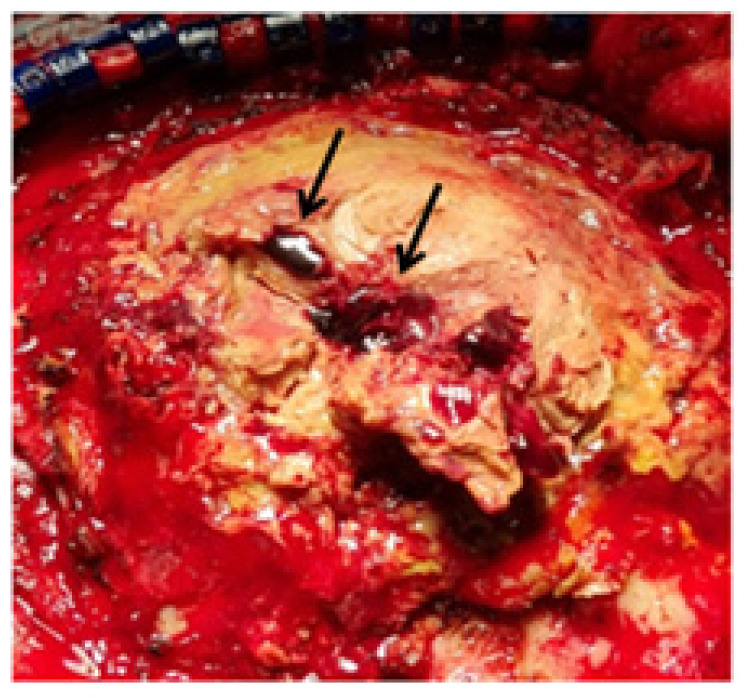
Intraoperative view shows a thick yellowish mass and microbleeds (arrows) occupying the epidural space. Subsequently, aggressive debridement and copious irrigation with autologous bone flap removal were performed. During the surgery, a white-yellow tissue layer was observed on the surface of the dissolved cranioplasty material. After removal of the resorbed autologous bone, an organized, bright brown and rigid abscess mixed with a hemorrhagic component was observed (Figure 5). The epidural space was sufficiently washed out using vancomycin mixed with saline irrigation. After surgery, a full course of systemic antibiotics was administered according to the recommendations of an infectious disease consultant, and the patient was transferred to the internal medicine department for further antibiotic treatment. The postoperative course was uneventful. On postoperative day 2, he was alert and started to speak with normal articulation, and his speech was completely normal on day 7. The differential diagnosis for transient aphasia is ischemic and hemorrhagic strokes, neoplasms, cerebral abscesses, and traumatic brain contusions [3]. In our case, the purulent discharge culture was negative before surgery, as well as during the removal of autologous bone graft with surrounding reactive tissues; however, we eventually diagnosed the case as a delayed infection after cranioplasty. Our patient also had a normal body temperature, normal WBC count, and normal levels of infection markers, such as ESR and CRP. The most reliable indicators of delayed infection after cranioplasty are the clinical features of the patients and imaging studies as well as laboratory results. We report a rare case of delayed cranioplasty infection that presented as aphasia 6 years after the cranioplasty. This report suggests that the possibility of such delayed infection should be considered. Furthermore, surgical debridement and bone flap removal for delayed infection should be conducted as soon as possible even when a patient presents with subtle infectious signs.
